# Non-Tuberculous Mycobacteria Interference with BCG-Current Controversies and Future Directions

**DOI:** 10.3390/vaccines8040688

**Published:** 2020-11-16

**Authors:** Deepshikha Verma, Edward D. Chan, Diane J. Ordway

**Affiliations:** 1 Department of Microbiology, Immunology and Pathology, Mycobacteria Research Laboratory, Colorado State University, 1682 Campus Delivery, 200 West Lake Street, Fort Collins, CO 80523, USA; Deepshikha.Verma@colostate.edu; 2 Department of Medicine, Rocky Mountain Regional Veterans Affairs Medical Center, Denver, CO 80523, USA; ChanE@NJHealth.org; 3 Division of Pulmonary Sciences and Critical Care Medicine, Departments of Medicine and Academic Affairs, National Jewish Health, University of Colorado Anschutz Medical Campus, Denver, CO 80523, USA

**Keywords:** *Mycobacterium tuberculosis*, non-tuberculosis mycobacteria, Bacille Calmette-Guérin

## Abstract

The global tuberculosis (TB) epidemic caused by the bacterial pathogen *Mycobacterium tuberculosis* (*M.tb*) continues unabated. The *Mycobacterium bovis* bacillus Calmette–Guérin (BCG) vaccination is widely utilized worldwide to protect against infection with *M.tb*. BCG vaccine protection against TB has had widely varying results for reasons that are not well understood. BCG vaccine interference by non-tuberculosis (NTM) mycobacterial species has been implicated as the potential cause of reduced BCG vaccine efficacy against *M.tb*. Ongoing efforts to develop new vaccines for TB requires a thorough understanding of the effect of NTM exposure on BCG vaccine efficacy, which may ultimately be a critical determinant of success. We reviewed the conflicting reports on whether NTM interferes with the BCG vaccine, potential explanations to help resolve the controversy, and strategies for developing better animal models. Further studies are needed to longitudinally track the effects of NTM exposure on BCG vaccine-induced host-protective anti-TB immunity.

## 1. Introduction

Tuberculosis (TB), caused by *Mycobacterium tuberculosis* (*M.tb)*, remains the leading cause of death by an infectious agent worldwide, resulting in 10 million new cases and 1.6 million deaths per year [1]. Mycobacteria are capable of surviving in intracellular compartments in phagocytic cells [2]. Immunity against *M.tb* requires a balance between immune responses that kill or at least constrain mycobacterial replication [3] and immune regulation to dampen tissue-damaging inflammation. One example that strongly supports the importance of a protective cellular response against *M. tb* [4,5] is individuals with the acquired immunodeficiency syndrome (AIDS), who have increased susceptibility to *M. tb* due to a deficiency of CD4+ T cells [4,6]. Before the development of the highly-active anti-retroviral cocktail therapies that are currently used against HIV infection, disseminated infections due to the non-tuberculous mycobacteria (NTM), particularly with *Mycobacterium avium* complex (MAC), were relatively common, further revealing the importance of the CD4+ T cells in host defense against mycobacteria [7,8]. However, with the development of an effective anti-HIV drug cocktail and restoration of the immune system, disseminated MAC infections are rarely seen in this population. Treatment of concomitant TB and HIV remains problematic due to the drug–drug interactions of rifampin with certain anti-HIV drugs. Recently, rifampin was shown to be used more frequently for TB treatment than *M. avium* treatment. Clinical trials have demonstrated that adding rifampin to the treatment regimen for *M. avium* did not improve MAC patient outcomes [9].

Regulatory T cells (Tregs) express the transcription factor Foxp3+ and are essential to counterbalance any excessive pro-inflammatory Th1 responses as the *M.tb* infection is being brought under control. Foxp3+ Tregs can quickly expand, but if they do so too early in the course of the *M.tb* infection, they may impede the onset of host-protective cellular immunity. Thus, it is not surprising that Tregs have been found to persist during chronic *M.tb* infection [3]. In experimental animal models, increased Treg response has been associated with a detrimental outcome of active *M.tb* infection, but this depended on the *M.tb* strain, animal model, and the stage of infection [10]. The ability of the host to temporally regulate the Treg number and activity helps dictate whether there is control of the infection or an uncontrolled progressive disease. Thus, while an early and over-exuberant Treg response may impair host control of an *M. tb* infection [3,11], a later inadequate Treg response may result in excessive inflammation and tissue injury as has been described in NTM-infected cystic fibrosis patients [12,13].

Bacillus Calmette–Guérin (BCG) is a live attenuated strain of *Mycobacterium bovis* currently used as the only vaccine against TB. The goal of vaccination is to establish a stable population of long-lived memory T cells. The BCG vaccine has been widely used in many parts of the world—but not the U.S., Canada, or several European countries—for well over 60 years to protect against meningeal TB in infants in TB-endemic countries [14]. However, BCG is unreliable to fully protect against pulmonary TB in adults, with studies showing widely disparate rates of protection [15]. Several theories have been proposed on why such a range of efficacy has been seen with BCG, including (i) the theoretical concern of genetic variation in the BCG strains used due to hundreds of passages and differences in BCG growth protocols employed, (ii) high background prevalence of a non-protective Th2 response (that may antagonize the desired immune response to BCG) in areas of the world endemic for helminths—which may be related to reduced efficacy in countries closer to the equator), (iii) waning BCG-induced immunity with time, (iv) less of a response to BCG in older individuals, (v) socioeconomic/psychosocial factors and nutritional status, and (vi) interference of BCG-induced immunity by NTM [16]. Studies in 2020 have illustrated that prior sensitization with NTMs could stop BCG multiplication and thereby prevent the induction of an effective BCG immune response and, ultimately, protection against TB infection [15,17]. Furthermore, different NTM species isolated from soil and sputum in Karonga, Malawi, an area where BCG has been shown to provide no protection against TB, differed in their ability to inhibit BCG multiplication [18,19]. In contrast, a TB subunit vaccine had the same protective effect in naive animals and those sensitized to NTMs [20,21]. Since the species of environmental NTM may vary widely in different regions and countries, the overall impact on BCG-induced immunity remains unknown. For example, in 2008, an Nontuberculosis Mycobacteria Network European trials group (NTM-NET) collaborative study obtained pulmonary NTM isolates and their identifications from laboratories in different regions of the world to gain further insight on the geographical distribution of NTM species cultured from respiratory samples at a single time point. These findings demonstrate the variations of NTM found in different regions of the world. Overall, the most abundant NTM isolated from pulmonary NTM patients were MAC species, followed by *M. kansasii*, *M. xenopi*, *M. malmoense*, *M. abscessus*, and *M. fortuitum* [22].

More recent studies have shown that BCG given twice rather than once may provide longer-lived BCG vaccine-induced protective immunity [23]. Indeed, there are some countries in which children are given multiple BCG vaccines; e.g., Turkey gives BCG vaccination at five different times during childhood: At birth, at two months after birth, at six to seven years of age, at eleven to twelve years of age, and at sixteen to seventeen years of age [24]. Whether this is the reason for the incidence of TB in Turkey being generally lower than its neighboring countries is not known [25]. In India, BCG vaccine is given once within one week of childbirth. The TB incident in India is extremely high and this supports the idea that multiple BCG vaccinations may result in a longer duration of protective immunity against TB. Recently, it was shown that BCG given intravenously to nonhuman primates afforded much greater protection against subsequent *M.tb* challenges as compared to the intradermal or aerosol delivery of the BCG [25].

We review below what is known about the epidemiological and clinical aspects of individuals infected with NTMs to identify the main modes of exposure to NTM, intending to shed light on how NTM may interfere with BCG efficacy. We also summarize the host immune deficits predisposed to isolated NTM lung disease or disseminated NTM disease. We then consider how these factors, such as the route of NTM exposure, the paradigm of repeated NTM infections, and the type of animal model, may result in the ability of NTM to interfere with the BCG vaccine. Insights into the differences of how humans compared to animal models respond immunologically to BCG vaccination and NTM interference studies are reviewed in the context of differences in clinical aspects, immune regulation, and existing gaps in knowledge and future directions.

## 2. Epidemiological and Clinical Aspects of Non-tuberculosis Mycobacterial Infection and Disease

NTM are mycobacterial species other than the *M.tb* complex and *Mycobacterium leprae*. While NTM are generally considered to be less virulent than *M.tb*, they can cause pulmonary and extrapulmonary disease in vulnerable individuals [26]. The initial methods for identification of NTM were developed by Ernest Runyon in the 1950s, in which species classification was based on growth characteristics such as slow-growing or rapid-growing and the absence or presence of colony color. The development of 16S rRNA gene sequencing and even more species-specific gene sequencing (such as of *rpoB* and *hsp65* genes) has replaced these methods and allowed for the current identification of approximately 200 NTM species. Improved methods since that time are currently whole-genome sequencing, allowing for accurate identification and epidemiological studies [27].

The prevalence of pulmonary NTM lung disease is overall increasing globally and has been attributed to many factors, including the greater prevalence of NTM in the environment due perhaps to global warming, greater exposure to NTM in the home environment due to increased use of showers for bathing, increased exposure to the outdoor environment from recreational activities, environmental disruption due to natural disasters, the aging population in many countries, and increased diagnosis due to greater use of chest CT scans that trigger search for NTM [23]. In addition, improved methods of diagnosis, such as fluorescence hybridization assay using peptide nucleic acid probes for identification and differentiation of tuberculous and NTN, and line probe assays (LPA) [28] may, in part, have contributed to the greater prevalence observed by increasing the sensitivity of the diagnostic tests. Geographic areas with humid climates appear to increase the prevalence of NTM disease, such as in Taiwan, where a surge of *M. abscessus* infections was seen, and in the Hawaiian Island environment. [29,30]. Hawaii has been studied as a model to identify other sources of exposure as it is the U.S. state with the highest number of NTM lung disease cases. In addition, mixed NTM infections have occurred involving MAC species, *M. abscessus*, and/or *M. kansasii*, and have been associated with worse clinical outcomes [31].

More recent studies have evaluated pulmonary lung infections in cystic fibrosis patients with *M. abscessus*, a species of multidrug-resistant non-tuberculous mycobacteria [32]. Cystic fibrosis patients infected with *M. abscessus* experience accelerated lung damage, causing increased morbidity and mortality. *M. abscessus* was thought to be acquired by susceptible individuals from the environment. However, new studies using whole-genome analysis of a global collection of clinical isolates demonstrate that most *M. abscessus* infections are not acquired through the environment but are acquired by transmission, potentially via fomites and aerosols. These transmitted fomites and aerosols of recently emerged dominant circulating clones have spread globally. We demonstrate that these clones are associated with worse clinical outcomes, showing heightened virulence in cell-based and mouse infection models. These studies indicate that if a healthy human’s upper respiratory tract can be colonized with NTMs and persist, the potential of household transmission may occur. The number of inhaled NTMs and independent exposures required for the establishment of lung disease remains unknown. Studying whether reinfection plays a potential role in the pathogenesis of NTM lung disease is highly relevant since repeated infections most likely occur given the ubiquitous nature of NTM in the environment. The missing gaps of knowledge in understanding the process of colonization, reinfection, and disease progression of NTM-infected patients who are then infected with TB also remains unknown.

The majority of healthy humans exposed to NTM through an aerosol or drinking water and through immune control of NTMs do not become infected nor show clinical symptoms through the stomach route [33]. However, individuals with gastric diseases such as gastritis, peptic ulcers, and gastric cancer have colonization of *M*. *abscessus* [34]. Before the development of more effective anti-HIV treatment, the gut appeared to be one of the major portals of *M. avium* infection in AIDS patients with disseminated NTM disease [35]. It has been established that murine autoimmune deficiency syndrome (MAIDS) virus infection or direct depletion of CD4+ cells substantially reduced overall T cell responsiveness and NTMs persisted in the stomach of MAIDs mouse models [35]. The pH in a healthy human stomach can be very low, around pH 2, which will kill NTM [36]. Furthermore, this is supported by the finding that one risk factor for acquiring NTMs is taking proton pump inhibitors [37], which increases the pH of the stomach.

**Figure 1:**
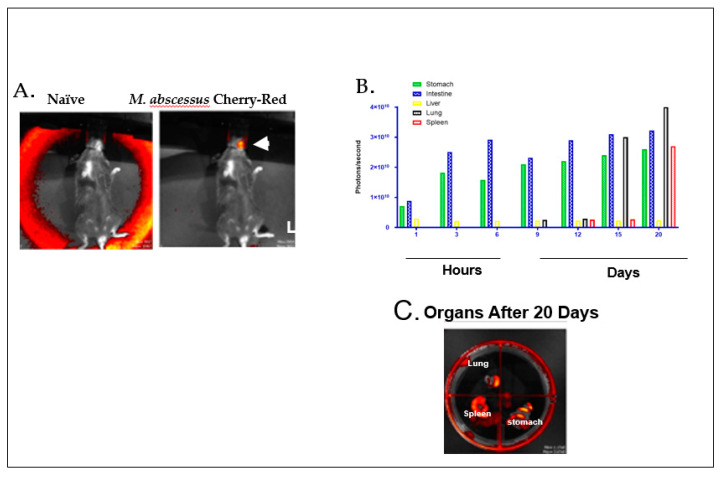
(**A**) C57BL6 mice infection by gavage with *Mycobacterium abscessus* OM194 strain expressing fluorescent marker in cherry-red. Infection in naïve shows no infection (red, white arrow). (**B**) Dissemination of *M. abscessus* OM194 strain mainly in the lung and spleen after 20 days of oral gavage infection. (**C**) Organs excised from the mouse after 20 days can individually receive xenogeny imaging for quantification of the bacterial burden expressions photon/second.

In contrast, the stomach pH of murine models range from 3.0–4.0, which allows a greater chance of NTM delivered through drinking water to survive [35]. In fact, Figure 1 demonstrates gavage infection of red fluorescent protein-tagged *M. abscesses* administered through drinking water to immunocompetent mice, which demonstrated direct infection of the stomach, intestines, lung, spleen, and liver. This murine model has been used to study BCG vaccination, and subsequent NTM interference allowing for TB disease progression is not an ideal model. The route of NTM administration does not mimic the aerosol route acquired in human disease.

NTM infections can be broadly divided into those that cause skin and soft tissue infections (due most often to the seeding of NTM from accidental trauma or iatrogenic infections), isolated NTM lung disease (likely the most common form and most often due to underlying structural lung diseases such as emphysema or pre-existing bronchiectasis), or disseminated disease (occurring in those with immunodeficiency) [26,38]. The two major radiographic phenotypes of NTM lung disease are the nodular-bronchiectasis and fibrocavitary forms, which have differing lung pathology and bacterial burdens [39]. The nodular-bronchiectasis form is most common in immunocompetent hosts, perhaps more common in women, and associated with granuloma formation in the airway walls and lower bacterial burden [40]. The upper lobe fibrocavitary pattern of NTM lung disease typically occurs in men with underlying emphysema and is generally associated with a greater bacterial burden and worse clinical outcome [41]. It must be noted that each type is not exclusively seen in male or female patients, and both types can be evident in a single patient [42]. Additional host factors associated with NTM lung disease include gastroesophageal reflux, advanced age, thin body habitus often with thoracic cage abnormalities such as scoliosis and pectus excavatum, and the use of inhaled corticosteroids and anti-tumor necrosis factor-alpha (anti-TNF-α) therapies [32,43].

## 3. Host Immune Defects in NTM Infection and Progressive Disease

Ongoing efforts to improve BCG or to develop new vaccines for TB require an understanding of the effect of NTM on vaccine efficacy. They are a critical determinant of success [44]. One approach to understanding the impact of NTM on the host is to look at the susceptible cohorts of individuals who become infected with NTM, display progressive disease, and may ultimately succumb to it. This allows us to track the main NTM routes of infection, sites of disease, host susceptibilities, and efficacy of BCG or any potential new vaccine that may afford protection against TB. Individuals with certain acquired or genetic/heritable disorders that compromise the lung architecture or lung immune system are more vulnerable to NTM lung disease. Some of the more well-accepted acquired disorders include smoking-related emphysema, bronchiectasis as a sequela of prior unrelated infections, pneumoconiosis such as silicosis, chronic aspiration, and use of corticosteroids, and other immunosuppressive agents such as TNF-alpha antagonists.

Individuals with genetic/heritable disorders in which bronchiectasis, emphysema, and/or lung immune defects are important sequelae are also predisposed to NTM lung disease; such underlying disorders include cystic fibrosis (CF), primary ciliary dyskinesia, alpha-1-antitrypsin deficiency, congenital bronchial cartilage deficiency (Williams–Campbell syndrome), tracheobronchomegaly (Mounier–Kuhn syndrome), Sjogren’s syndrome, pulmonary alveolar proteinosis, and common variable immunodeficiency [38]. It is important to emphasize that acquired and genetic/heritable predispositions are not mutually exclusive. Thus, it is a plausible supposition that the more risk factors an individual has, the greater the likelihood of developing NTM lung disease.

The occurrence of NTM lung disease in individuals without any of the aforementioned identifiable host risk factors is well recognized [42,45]. Enduring clinical experience has noted that a significant number of such patients possess a life-long slender body habitus with thoracic cage abnormalities such as pectus excavatum and scoliosis, leading to the notion of an underlying syndrome—perhaps a connective tissue disorder—that predisposes to NTM lung disease [38].

Another, non-mutually exclusive possibility in these thin individuals is that reduced body fat in itself is a risk factor for NTM lung disease [38,39,46]. One proposed mechanism by which low body fat content may predispose such individuals to NTM lung disease is a relative deficiency of the fat-derived, satiety hormone, leptin, since leptin also drives the differentiation of uncommitted T_0_ cells toward the T_H_1, interferon-gamma (IFNy)-producing phenotype [47]. This hypothesis is corroborated by the finding that leptin-deficient mice are more susceptible to *M. abscessus* experimental lung infection [38] and that NTM lung disease patients have reduced serum leptin levels [48] or a loss in the normal direct relationship between serum leptin concentration and total body fat [39].

A more recent whole-exome sequencing study of patients with NTM lung disease and their family members indicates that possessing variants of several genes in the immune, connective tissue, ciliary, and CF transmembrane conductance regulator (CFTR) categories—“multigenic” in nature as opposed to mutation of one dominant gene—may additively increase vulnerability to NTM lung disease [49]. Becker and colleagues [50] performed whole-exome sequencing on 11 NTM lung disease subjects with slender body habitus, pectus excavatum, and scoliosis and found four (two being sisters) with heterozygous mutations of the *MACROPHAGE-STIMULATING 1 RECEPTOR* (*MST1R*) gene and in none of 29 NTM lung disease patients without pectus excavatum or scoliosis. Significantly, MST1R is a tyrosine kinase receptor important for normal movement of cilia present on cells lining the luminal surface of fallopian tubes and airways [50]. Reduced ciliary movement due to a defect in MST1R function is consistent with previous work showing reduced ciliary beat frequency in the nasal epithelium and reduced nasal nitric oxide (NO) in NTM lung disease patients compared to controls [51].

While frank deficiency of IFN-y (e.g., advanced AIDS) or its upstream or downstream signaling molecules predisposes to extrapulmonary visceral/disseminated NTM disease, several studies have found reduced IFN-y production from the peripheral blood mononuclear cells (PBMC) or whole blood from NTM lung disease patients [52,53], acknowledging that this is not seen by others [54]. More recently, decreased IFN-y gene expression in the whole blood of NTM lung disease patients compared to control subjects who had bronchiectasis/chronic obstructive pulmonary disease but no NTM infection. While the mechanism(s) for reduced IFN-y is likely to be multifactorial, MST1R may participate in increasing IFN-y production by PBMC in addition to its role in maintaining normal ciliary function [50].

Patients with extrapulmonary visceral organ/disseminated NTM disease almost always have immunodeficiency. Acquired disorders that give rise to such profound immunodeficiency include untreated AIDS, the use of potent immunosuppressives for inflammatory diseases, cancer, or organ transplantation, and acquired autoantibodies to IFN-y [37,55]. One caveat is that there is likely a genetic component to those with anti-IFN-y antibodies as this syndrome appears to be more common in Asians and those with HLA DRB16:02 or DRB05:02 [56]. The occurrence of extrapulmonary visceral/disseminated NTM infections in very young individuals—often infants—should give concern for mutations of genes that are components of the IFN-y-interleukin-12 (IL-12) axes and other immune-related genes that fall under the rubric of mendelian susceptibility to mycobacterial diseases (MSMD) [38]. Susceptibility to NTM of individuals with MSDS was experimentally corroborated by the increased vulnerability to *Mycobacterium abscessus* in the IFN-y-knockout mice [57]. In adult individuals who develop extrapulmonary visceral organ/disseminated NTM disease but without acquired risk factors, the “MonoMAC syndrome” due to mutation of GATA2 transcription factor should be considered [58].

Some MSMD disorders—e.g., due to mutations of the genes for *RAR-related orphan receptor C* (*RORC*, a transcription factor essential for differentiation of T cells to the T_H_17 phenotype), *ISG15* (expected to reduce IFN-y production by T and NK cells), and interferon regulatory factor-8 (IRF-8, a transcription factor that regulates the differentiation of phagocytes as well as the production of IL-12 and TNF-α induced by lipopolysaccharide and IFN-y)—have not been reported to be associated with NTM infections; rather, such affected individuals have been afflicted with disseminated BCG infection [59,60]. However, the absence of reports of NTM infections in individuals with these rare genetic defects may simply be due to happenstance (the low likelihood of co-occurrence of an uncommon (NTM) disease with a rare disease), the failure to recognize the presence of an underlying genetic disorder even when such infections are present, and/or the lack of reporting of such cases even if identified; in other words, the absence of evidence of an association between NTM infection and a specific MSMD disorder does not necessarily mean evidence of absence of such a risk. While only three cases of NTM infections in chronic granulomatous disease (CGD) patients have been reported in the literature, the unusual and extrapulmonary involvement in these patients suggest that CGD is a predisposing condition for NTM infections: An *M. flavescens* disseminated infection, *M. fortuitum* pneumonia and osteomyelitis, and *M. avium* lung and mediastinal involvement in a ten month-old infant [61]. While these cases would suggest that reactive oxygen species (ROS) is an important host-defense factor against NTM infections, we have found that inhibiting ROS with a superoxide dismutase mimetic actually improved macrophage killing of *M. abscessus* by promoting phagosome–lysosome fusion [62].

## 4. Evidence for NTM Interference with BCG Efficacy

It is well established that the protective efficacy of BCG varies depending on which geographical region it is administered, and we know very little about why it protects when it does or why it fails to protect when it does not. Although a lot of progress has been made, the past two decades have revealed that there is no correlate of BCG-induced protection against TB even though evidence supporting T-cells, IFN-γ, TNF-alpha, and humoral immunity are clearly required [63].

**Figure 2:**
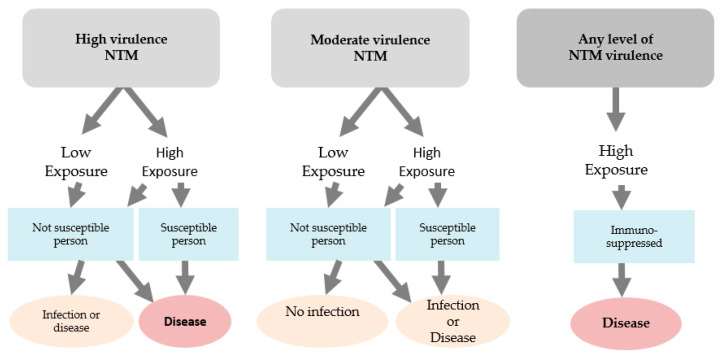
A hypothetical model explaining the persistence and/or reinfection of Nontuberculosis Mycobacteria (NTM) and the establishment of *Mycobacterium bovis* bacillus Calmette–Guérin (BCG) vaccine interference. Mouse models clearly indicate that TH1 immunity predominates in situations where this pathogen can be cleared, but there is little evidence, explaining its survival and chronic disease. One hypothesis involves T cell plasticity. The inflammatory Th17 response becomes dominant, resulting in loss of a protective Th1 response and its replacement by cells that can continually drive low-grade inflammation.

In countries where TB is endemic, the BCG vaccine has been given within the first few weeks of life of an infant since 1921 to prevent tuberculosis [21]. After an infant is BCG vaccinated, it can be exposed to NTMs in the environment through aerosolized water sources and fomite transmission. However, most healthy infants who get exposed and most likely infected with NTMs do not acquire a progressive NTM disease. Most likely, this life-long colonization occurs in BCG-vaccinated individuals. Figure 2 shows how if, over one’s lifespan, immune deficits are evident due to smoking, proton pump inhibitors, corticosteroids, thoracic skeletal abnormalities, or HIV, then the scale tips in favor of NTM infection and possibly diminished BCG vaccine-induced efficacy [64]. On the contrary, if no immune deficit appears, life-long NTM colonization after BCG vaccination could lead to the scale tipping in favor of NTM-induced protection. *M. tuberculosis* and NTMs have comorbidities with similar host immune deficits of cellular immunity CD4+ and CD8+, TNF-alpha, and rheumatoid arthritis [65].

It must be noted that the majority of animal studies utilized to evaluate NTM interference with the efficacy of BCG vaccine were performed with live or heat-killed NTM administered via their drinking water. Furthermore, there is a lack of the ability to quantify accurately in real-time the burden of viable NTM in live animals that are exposed to daily NTM as NTM have the capacity to replicate and produce biofilms even in a medium essentially devoid of nutrients such as mouse water bottles [66]. Thus, depending on the circumstances and time of experimental exposure, the mice may be exposed to unrealistically high amounts of NTM, which may not translate to a realistic human level of exposure. This delivery mode of the NTM is to the stomach, not directly to the lungs [67]. While the normal human stomach is more likely to be a barrier to NTM infection due to the presence of very low pH, it was shown that virulent *M. avium* strains could infect mice orally and be found in gut lymphoid tissues, perhaps due, in part, to greater pH in the normal murine gastric fluid. However, if beige mice with C57BL/6 background were used, the degree of infection was further increased [68,69]. Additional studies showed that *M. avium* accessed the gut epithelium by interactions with enterocytes [69]. The obvious issue with using this murine experimental plan is the main route of NTM transmission, and infection and disease occur through aerosol exposure. The majority of NTM infections are manifested as an isolated pulmonary disease [59]. It would be optimal to have future models developed to evaluate the impact of aerosolized NTM on BCG vaccine or novel vaccine efficacy and *M.tb* disease outcome.

The idea that NTM could interfere with BCG vaccination was supported by studies in which mice were immunized with NTM before BCG vaccination, and efficacy was subsequently reduced [70]. Recent unpublished studies in our laboratory indicate that exposure in the gut to *M. avium* induces regulatory T cells that counterbalance BCG-induced effector cells in the lungs after a challenge. A subsequent study highlighted that NTM could, in some cases, block effector immunity, diminishing protection by BCG, or could add to it. It is feasible that NTM generating T cell immunity results in a cross-reactive response to *M. tuberculosis* antigens, and this can reduce the efficacy of BCG by itself. Recent studies have shown that people exposed to NTM have IFN-γ T cells that recognize multiple proteins from the DosR gene complex [71]. Th1 immunity can be triggered through Toll-like receptors (TLRs), and it was found that mice lacking TLR2 were more susceptible to *M. avium* infection [72]. Chronic infection of mice can not only [73] result in severe necrosis, but can also (unlike tuberculosis) result in a gradual loss of T cells lymphopenia [74]. This also shows that the pathogenesis of NTM diseases is still less well understood, with elements of the disease process in response to certain NTM infections showing differences. Although multiple virulence factors associated with the NTM have been proposed to interfere with BCG efficacy, additional studies are required to understand NTM BCG vaccine interference.

## 5. Conclusions

The existing gaps in knowledge and future directions are many. For example, the vast majority of conflicting BCG vaccine interference with NTMs and *M. tuberculosis* has been derived from mouse models with the NTM bacilli persisting in the stomach. New models in which NTMs do not persist in the stomach should be developed, such as the guinea pig and nonhuman primate model of BCG vaccination and the NTM interference. Clearly, the best course is to use the human studies and animal models that best reproduce the target population, which is humans. The mouse models that have been completed have led us, for the most part, on a pathway of erroneous information. The primary model which can be developed and mimic humans is the guinea pig and NHP model, which has a low stomach pH, meaning NTMs do not survive, and primary and secondary granulomas, which are similar to humans and the NHP model. In summary, the scientific community must use models that represent not only NTM infection and disease, but also the knowledge of how BCG vaccination, NTM persistence, and TB outcome impact the human host.
